# Experimental Investigation on the Mechanical Behavior of Bovine Bone Using Digital Image Correlation Technique

**DOI:** 10.1155/2015/609132

**Published:** 2015-02-15

**Authors:** Yuxi Chen, Diansen Yang, Yongshang Ma, XianJun Tan, Zhan Shi, Taoran Li, Haipeng Si

**Affiliations:** ^1^Shandong Experimental High School, Jinan, Shandong 25001, China; ^2^State Key Laboratory of Geomechanics and Geotechnical Engineering, IRSM, Chinese Academy of Sciences, Wuhan 430071, China; ^3^Qilu Hospital of Shandong University, Jinan, Shandong 250012, China

## Abstract

In order to understand the fracture mechanisms of bone subjected to external force well, an experimental study has been performed on the bovine bone by carrying out the three-point bending test with 3D digital image correlation (DIC) method, which provides a noncontact and full field of displacement measurement. The local strain and damage evolution of the bone has been recorded real time. The results show that the deflection measured by DIC agrees well with that obtained by the displacement sensor of the mechanical testing machine. The relationship between the deflection and the force is nearly linear prior to reaching the peak strength which is about 16 kN for the tested bovine tibia. The full-field strain contours of the bone show that the strain distribution depends on not only the force direction, but also the natural bone shape. The natural arched-shape bovine tibia bone could bear a large force, due to the tissue structure with high strength, and the fracture propagation process of the sample initiates at the inner side of the bone first and propagates along the force direction.

## 1. Introduction 

Predicting and preventing bone fractures are an important topic in orthopaedics due to their high frequency, surgical complications, and socioeconomic impact [1]. Bones fracture when an accidental force exceeds the physiological range, inducing stresses over the strength. Fracture could be related to an external sudden high force or an internal degradation of bone strength caused by an osteoporosis disease. The first type of fractures often appears in athletes and the second type of fractures occurs mainly in aged people having osteoporosis. The mechanical properties of bone including bone's stiffness and strength strongly depend on the composition, shape, and age. Experimental testing of bone is a direct method to study the mechanical behaviour of bone and quantify bone strength. Standard mechanical tests including uniaxial compression test and three-point bending test are often employed to describe the mechanical behaviour of bone. During the test, integral information of the tested bone such as the relationship between force and displacement can be obtained. As bone is a heterogeneous natural composite material, the mechanical behaviour of bone should be closely related to its microstructure. In the past decades, a lot of studies have focused on identifying local strains and damage of bone by means of different methods such as high-speed photography, scanning electronic microscopy, CT, and digital image correlation [2–7]. The earlier studies show that these methods are useful to investigate local strain of bone and 3D DIC is more convenient to be used during mechanical test. Among these methods, digital image correlation is a novel optical, noncontact measuring technique that is widely used in material sciences such as metals, polymers, composites, and biomaterials [8]. Using one or several high-resolution couple charged device cameras, DIC tracks the gray level distribution in subsets of images of the specimen's surface covered with natural contrast or painted random black-white speckle pattern to determine displacement and strain fields of the material under any type of loading. Relative to 2D DIC that determines the full-field strain of the material in the plane with one camera, 3D DIC employs two cameras to create a stereo image and determine the three-dimensional coordinates of any surface point in space. 3D DIC can avoid the effect of out-of-plane motion which changes the magnification and introduces errors in 2D measurement [9]. In order to well understand the fracture mechanism of bone, 3D DIC method has been applied to study the mechanical behavior of natural bovine bone with three-point bending test.

## 2. Material and Methods 

### 2.1. Sample Preparation

**Figure 1:**
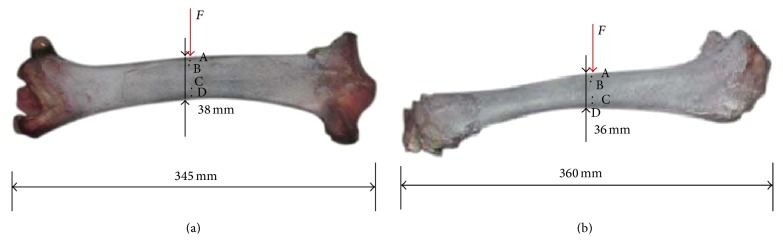
Photo of the two fresh bovine bone samples ((a) sample number 1; (b) sample number 2).

**Figure 2:**
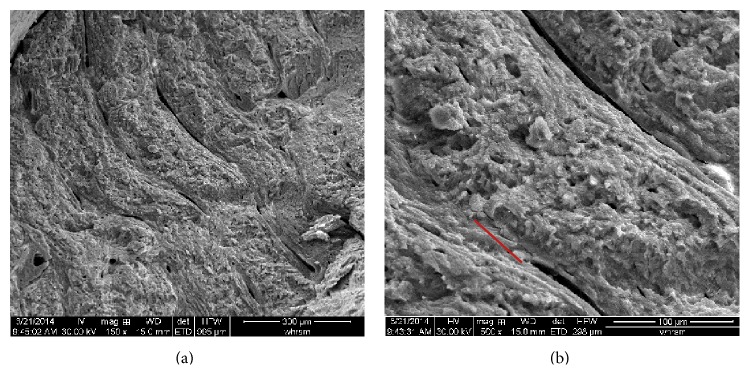
Microstructure of the bovine bone.

To study the mechanical behaviour of bovine bone, two fresh tibias from a male bovine 6 years of age were obtained from a butcher's shop. Prior to testing the leather and the muscle covered on the bones were removed. The length of the bovine tibia is 345 mm for sample number 1 and 360 mm for sample number 2. The diameter of sample number 1 is about 38 mm. The natural bone is shown in Figure 1. Bone is a natural composite material with hierarchical organization at different scales [10]. The fracture mechanisms of bone should be closely related to its particular microstructure. Therefore, the microstructure of the bovine bone was firstly investigated using the scanning electronic microscopy (SEM).  Figure 2(a) shows the profile image of the bovine cortical bone which is a typical plexiform cortical bone tissue structure. The cortical bone is in the form of lamellar layers and lamellae are parallel and are arranged along the bone's circumference.  Figure 2(b) shows that the length of lamellae is about 110 microns and the width of lacuna is several microns.

### 2.2. 3D DIC

**Figure 3:**
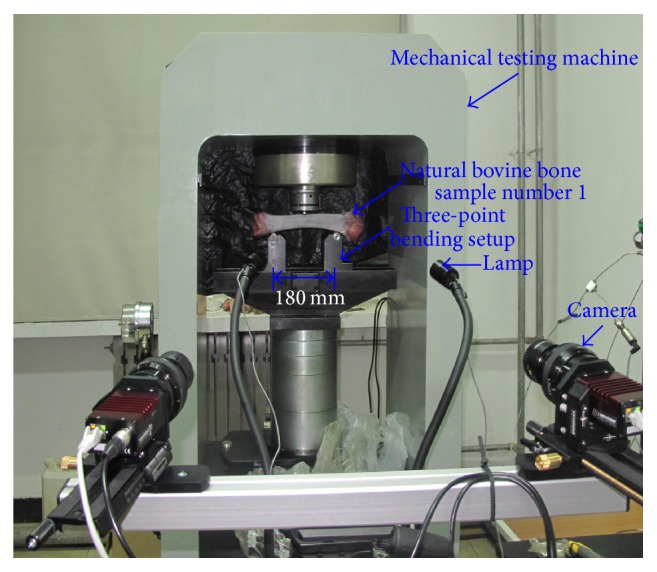
Photo of the three-point bending setup equipped with a 3D DIC system.

The 3D Digital image correlation (DIC) system, which uses two high resolution CCD cameras (AVT Prosilica GT with a resolution of 3384 × 2074 pixels), and a Rodagon 80 mm f/5.6 lens, is illustrated in Figure 3. The object distance is 600 mm. The angle between the optical axis of each camera and the initial normal to the specimen's surface is about 13°. This corresponds to an included pan angle between cameras of about 26°.

Firstly, system calibration was carried out before the test using various images of translated and rotated regular grid pattern within a bundle adjustment technique [8]. The initial 3D image of the bone was reconstructed by finding the corresponding speckles in the images captured by the two cameras using a triangulation method. Secondly, a similar procedure was applied to create the deformed 3D images. Finally, the deformed images were compared with the undeformed images and the 3D displacements and full-fields strain will be obtained. The strain measurements accuracy of this system is better than 10^−4^ at the macroscale (cm). Note that 3D DIC software is VIC-3D.

### 2.3. Mechanical Testing

In this study, the three-point bending test has been carried out using a specific setup adapted for a rigid mechanical testing machine. The support span in the three-point bending rig is 180 mm and the diameter of the indenter is 10 mm as shown in Figure 3. The two lower points of support are provided by a bovine bone. The third point of support is provided by an indenter, which exerts the force on the sample in the middle between the two lower points of support. The force is applied by the mechanical testing machine with either force or displacement controlling. In this study, the displacement control is chosen and the loading rate is 10 *μ*m/s. The whole test system including the three-point bending device, mechanical testing machine, and a dual-stereo-vision system, is illustrated in Figure 3.

A series of uniaxial compression tests have been also performed on several rectangular samples (24 × 16 × 6 mm) prepared from the same bovine bone to gain the compression strength of the bovine. During the test, the displacement control is chosen and the loading rate is 10 *μ*m/s.

## 3. Results and Discussion

The three-point bending tests performed on the bone samples number 1 and number 2 have lasted about one hour when the bone fractured. The images of the sample were captured with rate 10 per second simultaneously by two cameras and then analyzed using DIC technique to create the 3D images and identify the displacement and local strain of interested zones. For three-point bending tests, the middle part of the tibia is the most important part which has been treated by a speckle pattern of black spots on the surface as shown in Figure 1.

**Figure 4:**
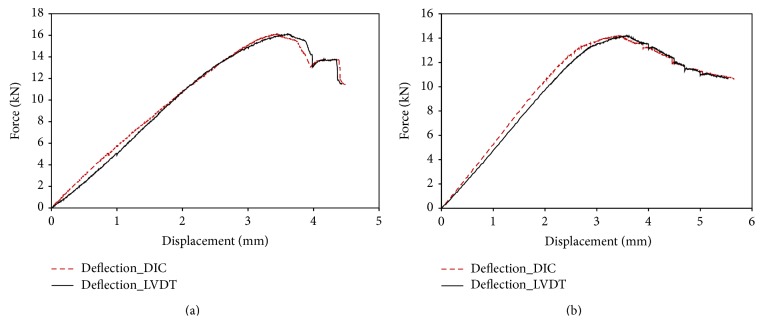
Deflection versus force curves of the two bovine bones during three-point bending test ((a) sample number 1; (b) sample number 2).

The deflection at the midpoint of samples number 1 and number 2 is illustrated in Figures 4(a) and 4(b), respectively.  Figure 4 shows that the deflection measured by DIC agrees well with that obtained by the displacement sensor LVDT of the mechanical machine. It confirms the performance of 3D DIC system. The deflection versus force curve is nearly linear before peak strength which is about 16 kN for sample number 1 and 14 kN for sample number 2. This linear behaviour should be related to the linear elastic behaviour of the material which is also observed during a series of uniaxial compression tests performed on several rectangular samples (24 × 16 × 6 mm) prepared from the same bovine bone. Young's modulus is determined to be 6.7 GPa in the long bone axis direction and 3.9 GPa in the radial direction. The measured Young modulus is relatively smaller than that observed on a frozen bovine femur by Szabó and Thurner in 2013 [11]. The difference could be related to the density of the bone. The postfailure curve is nonlinear and during this stage the strain continues to increase while the strength decreases; the phenomenon is strain softening and it indicates that the tibia is ductile. The strain softening should be firmly related to the initiation and propagation of fracture of the bones as shown in the strain contours (Figure 6).

**Figure 5:**
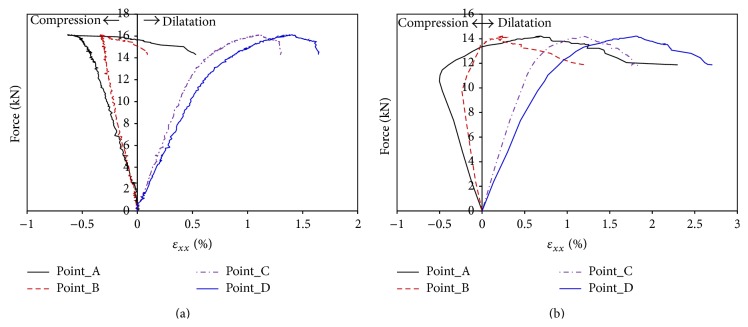
Strains versus force curves of three different zones ((a) sample number 1; (b) sample number 2).

**Figure 6:**
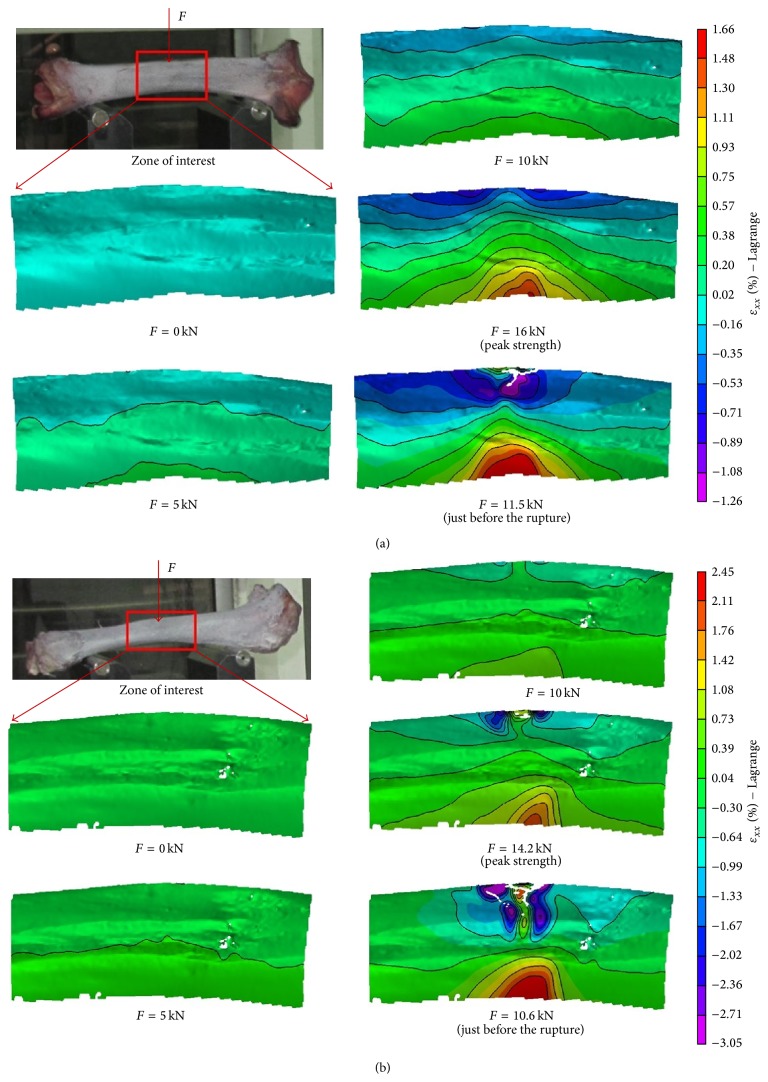
Strains map of sample number 1 (a) and sample number 2 (b) in the long bone axis direction during loading.

The local strain of the bovine tibia in the long bone axis direction of the 4 different points, which corresponds to an equivalent gage length of 0.24 mm, is illustrated in Figure 5. The results show that the base part (points C, D) dilates along the bone axial direction and the upper part (points A, B) near the indenter first contracts and then dilates when the force exceeds a level (16 MPa for sample number 1 and 11 MPa for sample number 2). This strain distribution is different from that observed by Szabó and Thurner [11] who showed that the strain of the rectangular sample prepared from a frozen bovine femur was always tensile during three-point bending test. The difference should be related to the sample size. The samples used by Szabó and Thurner in 2013 [11] are rectangular samples with small size (6 × 0.36 × 0.36 mm) and they are used to study the material properties, while the samples tested in this study are fresh bovine tibia with a length of 345 mm and a diameter of 38 mm and they are employed to study the mechanical behaviour of the whole bovine tibia. These results confirm that the mechanical behaviour of the bovine tibia depends on both the material properties and the structure of the bone. Since the bovine tibia has an arched shape, the upper part of the bovine tibia is dominated by the compression during the primary loading phase and it is gradually subjected to tensile stress with the increase of the force until the fracture of the sample during three-point bending test. The strain map of sample number 1 in the bone axis direction (Figure 6) gives evidence of the increase of tensile strain in the base part and the change of compressive strain to tensile strain in the upper part during loading. The strain distribution is closely related to the irregular shape of the bovine tibia.

The strain map shows that the damage of the bovine tibia initiates at the upper part contacted with the indenter and propagates along the force direction when the force exceeds the peak strength. However, Figure 5 shows that the maximum measured tensile strain of point A (0.5% for sample number 1 and 2.3% for sample number 2) is less than that of point D on the base part (1.65% for sample number 1 and 2.6% for sample number 2). In fact, due to the short duration of the failure phase and loss of image information, the tensile strain at point A at failure could not be obtained. Moreover, the strain measured by 3D DIC is the strain on the surface of the bone and the internal strain could not be directly obtained. The maximum tensile strain of the bovine bone failure varies between 10% and 23% [11] which is much larger than that obtained at the surface in this study. The damage should initiate at the inner side, because the fresh bovine tibia is a hollow structure and the tested bone is filled with bone marrow. Such structure results in a different fracture mode relative to a small cuboid sample made from bone. At the same time, the upper part is directly contacted with the indenter which will induce a stress concentration and lead to the fracture of the part near the indenter. Therefore, the damage and propagation of the fracture near the indenter should be firmly related to both the concentration of stress created during the test and the bovine tibia irregular shape. It is needed to note that the microstructure observation of the bone at the end of the test (Figure 2(b)) gives evidence of the open of tissue structure and confirms that the tensile damage is major failure model during the three-point bending test.

## 4. Conclusions

In order to study the mechanical behaviour of bovine bone, three-point bending tests have been carried out on fresh bovine tibias using 3D digital image correlation technique. The quasi-linear relationship between the deflection and the force is found while the force does not exceed the peak strength which is about 16 kN for sample number 1 and 14 kN for sample number 2, and the linear behaviour of the bovine tibia should be related to the linear behaviour of bone cell. The local strain of the bovine bone is quantified using 3D digital image correlation method and the results show that the strain distribution is related not only to the force direction, but also to the bone shape. The upper part of the arched-shape bovine tibia is dominated by compression during the quasi-linear phase and it is gradually subjected to tensile stress with the following nonlinear phase. This transition from compaction to dilation is mainly related to both the applied stress and the bovine tibia irregular shape. The full-field strain map obtained during the test and the microstructure observation of the bovine bone at the end of the test confirm that the tensile damage is the main failure mode during the three-point bending test.
